# Hepatoprotective Effect of Dried Red Jujube Fruit Extract Against Acetaminophen-Induced Acute Hepatotoxicity

**DOI:** 10.7759/cureus.33272

**Published:** 2023-01-02

**Authors:** Cecilia P Tedyanto, Laura Wihanto, Adi P Hendrata

**Affiliations:** 1 Department of Clinical Pathology, Faculty of Medicine, Widya Mandala Catholic University, Surabaya, IDN; 2 Department of Microbiology and Parasitology, Faculty of Medicine, Widya Mandala Catholic University, Surabaya, IDN

**Keywords:** acetaminophen, aminotransferases, acute hepatotoxicity, acetaminophen toxicity, ziziphus jujuba, dried red jujube fruit extract

## Abstract

Background

A liver injury could impair the integration of the body’s organ system, which may cause complications that can lead to death. The dried red jujube fruit extract has the potential to protect the liver from toxic substances through its antioxidant properties.

Aims

To determine and analyze the hepatoprotective effect of dried red jujube fruit extract on aminotransferase levels against acetaminophen-induced acute hepatotoxicity.

Methods

Male Wistar rats were divided into five groups. The negative control group (G1) received carboxymethylcellulose sodium (CMC-Na) 1%. The positive control group (G2) received acetaminophen. The treatment group G3 received dried red jujube fruit extract 70 mg/kg BW + acetaminophen, G4 received dried red jujube fruit extract 140 mg/kg BW + acetaminophen, and G5 received dried red jujube fruit extract 280 mg/kg BW + acetaminophen. Dried red jujube fruit extract was given for 10 consecutive days. Acetaminophen (3 g/kg BW) was given on the ninth day. Blood samples were collected, and aminotransferase levels were measured on the 11th day.

Results

Kruskal-Wallis comparison test showed significant differences (*p* < 0.01) between all groups on alanine aminotransferase (ALT; *p* = 0.003) and aspartate aminotransferase (AST; *p* = 0.001) levels. Mann-Whitney post hoc test showed significant differences (*p* < 0.01) between G2:G3, G2:G4, and G2:G5 groups on ALT and AST levels. Pearson correlation test showed a significant negative correlation (*p* < 0.01; *r* = -1) between all given doses of dried red jujube fruit extract on ALT (*p* = 0.000; *r* = -0.778) and AST (*p* = 0.000; *r* = -0.774) levels.

Conclusion

The dried red jujube fruit extract has a hepatoprotective effect on aminotransferase levels against acetaminophen-induced acute hepatotoxicity at 70 mg/kg BW, 140 mg/kg BW, and 280 mg/kg BW (the most effective dose), and there was a negative correlation between all doses and the aminotransferase levels.

## Introduction

The liver is both a gland and a solid organ with complex functions and essential roles in various physiological processes in the human body [1]. A fatal condition can occur due to liver injury. The liver injury will reduce productivity and quality of life. A damaged liver can also impair the integration of the body's organ systems, so it is possible to cause various complications, which can lead to death.

Hepatotoxicity is a condition in which the liver is harmed due to exposure to toxic chemicals or medications. Acetaminophen, also known as paracetamol, is one of the hepatotoxins that can cause liver injury when taken in high dosages [2]. It is frequently used as an antipyretic and analgesic in the community.

Acetaminophen is an over-the-counter (OTC) drug that can be easily obtained and consumed without supervision or even a prescription from a doctor. Acetaminophen is highly prone to be consumed impulsively due to inadequate monitoring and education, which might cause toxic effects. Acetaminophen-induced hepatotoxicity is the leading cause of acute liver injury in the United States, accounting for around 50% of all cases [3].

Serum glutamic pyruvic transaminase (SGPT), also known as alanine aminotransferase (ALT), is an enzyme that is quickly produced in cases of acute injury to liver cells or hepatocytes. ALT is a specific marker of hepatocyte injury because it exclusively exists in the cytoplasm of hepatocytes. Serum glutamic oxaloacetic transaminase (SGOT) or aspartate aminotransferase (AST) is also an indicator of acute hepatocyte injury. However, it is not a specific marker since AST is present in various organs in addition to the cytosol and mitochondria of hepatocytes [4].

Substances that play a role in protecting the liver from injury caused by hepatotoxins are called hepatoprotective drugs. Jujube fruit (*Ziziphus jujuba*) is one of the natural components that have the potential to be a future hepatoprotective drug. The varieties of jujube fruit currently available worldwide consist of fresh and dried fruit. Research has shown that both fresh and dried jujubes contain phenols and flavonoids that have an antioxidant capacity to minimize the injury of hepatocytes. The degree of maturity affects the total phenolic content (TPC) and total flavonoid content (TFC) of fresh jujube fruit. Yet, each maturity level still has higher amounts than the dried one [5,6].

Fresh jujube fruit has been shown to have various therapeutic effects, one of which is hepatoprotection [7]. Jujube fruit comes from China and is grown in temperate to subtropical climates [8]. Due to the tropical climate of Indonesia, the most frequent jujube fruit that we may find is the fruit that has reached the peak of maturity and has undergone drying, packaging, and product storage.

This research was conducted to prove that jujube fruit in Indonesia has or does not have a hepatoprotective effect because it has been dried, packaged, and stored during the distribution process. This research aimed to determine and evaluate the hepatoprotective effect of dried red jujube fruit extract on aminotransferase levels against acetaminophen-induced acute hepatotoxicity and to analyze the correlation between the dose of the extract and its hepatoprotective effect. This research can also support the development of dried red jujube fruit extract as a future hepatoprotective drug.

## Materials and methods

Plant materials

Dried red jujube fruits were purchased from a local herbal shop in Surabaya, Indonesia. The fruits were identified by the Bioscience and Plant Technology Laboratory, Sepuluh Nopember Institute of Technology, Surabaya, Indonesia.

Preparation of extract

Dried red jujube fruit seeds were removed, and the fruits were cut and dried using an oven at 40°C. The dried fruits were crushed using a grinding machine until they became a homogeneous powder. The powder was macerated with 70% ethanol for 24 hours. The maceration results were filtered to obtain the filtrate and residue. The residue was re-extracted in the same step three times. The combined extracts were evaporated using a rotary evaporator at a temperature of 40°C until a viscous mass was obtained.

Determination of phytochemical substances

The phytochemical substances were determined using color and precipitation reactions for each characteristic reagent. Alkaloids were identified by the Mayer and Dragendorff reagents, flavonoids were identified by the reaction with hydrochloric acid and magnesium powder, saponins were identified based on their foaming abilities after being shaken with hydrochloric acid, tannins were identified by ferric chloride, and triterpenoids were identified using the Liebermann-Burchard reagent.

Determination of total phenolic content

TPC was determined using the Folin-Ciocalteu colorimetric method. The absorbance was measured at 760 nm, and the average value of triplicate data was expressed in gallic acid equivalent (mg of GAE/g of extract).

Determination of total flavonoid content

TFC was determined in triplicate by the aluminum chloride colorimetric method. The absorbance was measured at 510 nm, and the results were expressed in rutin equivalent (mg of RE/g of extract).

Preparation of acetaminophen suspension

Acetaminophen has low water solubility. Therefore, a suspending agent (carboxymethylcellulose sodium or CMC-Na) is needed for the dilution process. Acetaminophen tablets were crushed using a mortar and pestle into a fine powder and then dissolved with a 1% CMC-Na solution until homogeneous.

Animals

Male Wistar rats (150-200 grams, 10-12 weeks old) were purchased from the Biochemistry Laboratory of the Faculty of Medicine, Hang Tuah University, Surabaya, Indonesia. Rats were medically checked by a veterinarian and declared healthy by the Department of Livestock East Java, Surabaya, Indonesia. All rats were acclimated under laboratory conditions for seven days and were maintained with standard chow and distilled water ad libitum. They were placed in groups in ventilated cages with wire top covers and a soft bed of husk at a controlled temperature (20-24°C), humidity (45-65%), and lighting (12 hours of light/dark cycle).

Ethical clearance

These research procedures complied with the ethical principles outlined in the Council for International Organizations of Medical Sciences (CIOMS) and World Health Organization (WHO) International Ethical Guidelines for Health-Related Research Involving Humans and CIOMS Geneva. The protocol was approved by the Health Research Ethics Committee (HREC), Faculty of Medicine, Widya Mandala Catholic University, Surabaya, Indonesia (protocol number: 0265/WM12/KEPK/MHS/T/2022).

Experimental design and protocol

Male Wistar rats were divided into five groups as follows: (1) negative control group or G1 (1% CMC-Na solution); (2) positive control group or G2 (acetaminophen); (3) treatment group 1 or G3 (dried red jujube fruit extract 70 mg/kg BW + acetaminophen); (4) treatment group 2 or G4 (dried red jujube fruit extract 140 mg/kg BW + acetaminophen); (5) treatment group 3 or G5 (dried red jujube fruit extract 280 mg/kg BW + acetaminophen). Dried red jujube fruit extract was given for 10 consecutive days. On the ninth day, acetaminophen (3 g/kg BW) was given. Intracardiac blood samples were collected on the 11th day, and aminotransferase levels were measured. Rats were anesthetized with ketamine at 100 mg/mL (50-100 mg/kg BW) by intramuscular injection in the caudal thigh muscle.

Blood collection

Surgery was performed until the heart was visible. The blood was drawn by cardiac puncture using a syringe with a fixation position forming a 45-degree angle to the heart organ. The blood in the syringe was put into a plain vacutainer tube labeled with the group code. Blood was allowed to clot for about 30 minutes; then, serum was separated by centrifugation at 3000 rpm for five minutes. The serum was used to assess the levels of ALT and AST.

Assessment of liver function

ALT and AST serum measurements were carried out by the enzymatic colorimetric method using a colorimeter (COBAS INTEGRA® 400, Roche Diagnostics, Basel, Switzerland). The aminotransferase levels were determined by measuring the decrease in absorbance at 340 nm. The results were expressed in IU/L.

Statistical and correlation analysis

The experimental results were presented as mean ± standard deviation (SD) and were analyzed by the Kruskal-Wallis test with the Mann-Whitney test as the post hoc test. The significance values (*p*) at *p* < 0.01 represent statistically significant differences. The correlation coefficient (*r*) for the aminotransferase levels and the levels of dried red jujube fruit extract was calculated using the Pearson test. The *r *values are considered as follows: -1 (negative correlation), 0 (no correlation), and +1 (positive correlation). The statistical and correlation analyses were carried out using Statistical Package for the Social Sciences (SPSS) version 25 (IBM Corp., Armonk, NY).

## Results

Phytochemical substances

The phytochemical screening of the dried red jujube fruit extract showed positive results on alkaloids, phenols, flavonoids, saponins, tannins, and triterpenoids (Table 1). The TPC of the dried red jujube fruit extract was 6.52 mg GAE/g, and the TFC was 1.92 mg RE/g (Table 2).

**Table 1:** Phytochemical screening results of dried red jujube fruit extract

Substance	Result
Alkaloids	Positive
Phenols	Positive
Flavonoids	Positive
Triterpenoids	Positive
Tannins	Positive
Saponins	Positive

**Table 2:** Total phenolic content and total flavonoid content results of dried red jujube fruit extract GAE: gallic acid equivalent; RE: rutin equivalent; g: grams.

Substance	Result
Total phenolic content (mg GAE/g)	6.52
Total flavonoid content (mg RE/g)	1.92

Levels of ALT and AST

**Figure 1:**
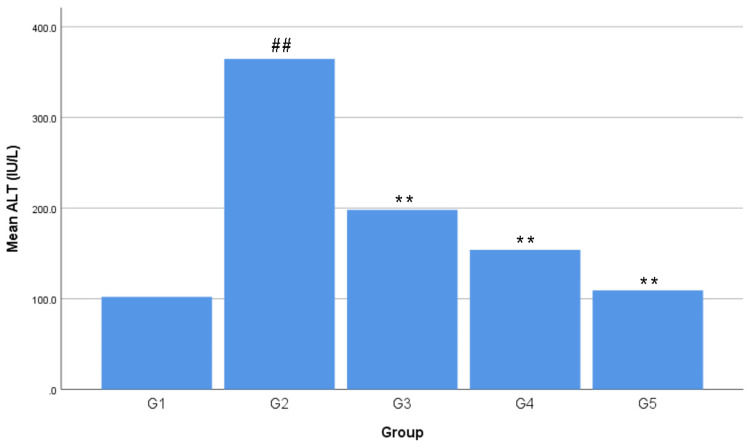
Effects of dried red jujube fruit extract on alanine aminotransferase (ALT) levels Data are expressed as mean ± standard deviation; n = five rats per group. ##: p < 0.01 vs. negative control group (G1). **: p < 0.01 vs. positive control group (G2).

**Figure 2:**
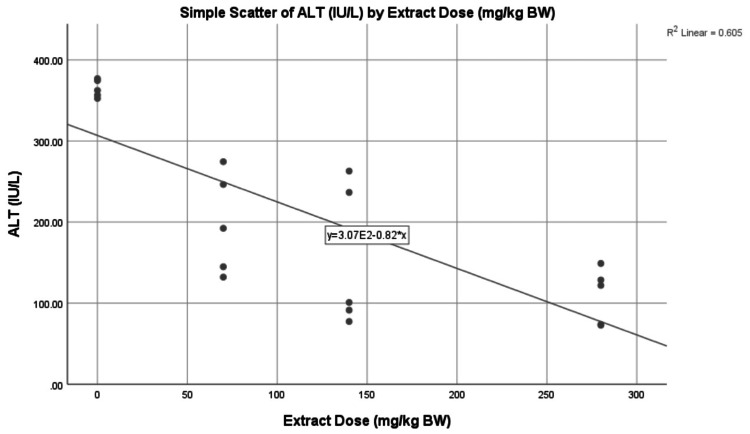
The correlation between extract doses and alanine aminotransferase (ALT) levels Scatterplot showing the negative correlation (p = 0.000; r = -0.778) between the dose of dried red jujube fruit extract and the ALT levels.

The lowest average ALT level was found in G1 with 102.08 ± 11.41 IU/L. The highest average ALT level was found in G2 with 364.56 ± 10.80 IU/L. The ALT levels in the treatment group formed a linear line between G1 and G2, sequentially from high to low, with an average G1 of 198.04 ± 62.03 IU/L, G2 of 153.84 ± 88.44 IU/L, and G3 of 109.22 ± 34.28 IU/ L (Figure 1). The average ALT level in G1 increased by 257.13% or +262.48 IU/L in G2, 94.01% or +95.96 IU/L in G3, 50.71% or +51.76 IU/L in G4, and 7% or + 7.14 IU/L in G5. The average ALT level in G2 decreased by 45.68% or -166.52 IU/L in G3, 57.80% or -210.72 IU/L in G4, and 70.04% or -255.34 IU/L in G5. The average ALT level in G3 decreased by 22.32% or -44.20 IU/L in G4 and 44.85% or -88.82 IU/L in G5. The average ALT level in G4 decreased by 29% or -44.62 IU/L in G5 (Table 3). There was a negative correlation between all extract doses and the ALT levels, forming a linear line from top to bottom, as seen in Figure 2. A negative correlation means the higher the dried red jujube fruit extract doses, the lower the ALT levels.

**Table 3:** Value difference and percentage of alanine aminotransferase levels G1: negative control group; G2: positive control group; G3: treatment group 1; G4: treatment group 2; G5: treatment group 3; SD: standard deviation; IU/L: international unit/liter; +/↑: increase; -/↓: decrease.

Group	Value difference (IU/L)	Percentage
G1 → G2	+262.48	↑ 257.13%
G1 → G3	+95.96	↑ 94.01%
G1 → G4	+51.76	↑ 50.71%
G1 → G5	+7.14	↑ 7%
G2 → G3	-166.52	↓ 45.68%
G2 → G4	-210.72	↓ 57.80%
G2 → G5	-255.34	↓ 70.04%
G3 → G4	-44.20	↓ 22.32%
G3 → G5	-88.82	↓ 44.85%
G4 → G5	-44.62	↓ 29%

**Figure 3:**
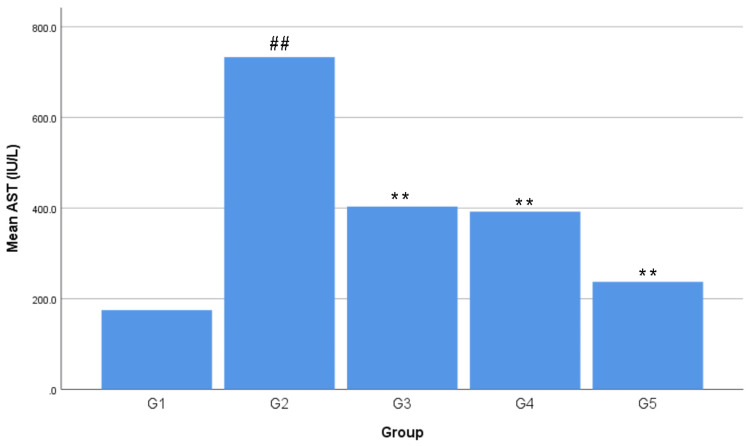
Effects of dried red jujube fruit extract on aspartate aminotransferase (AST) levels Data are expressed as mean ± standard deviation; n = five rats per group. ##: p < 0.01 vs. negative control group (G1). **: p < 0.01 vs. positive control group (G2).

**Figure 4:**
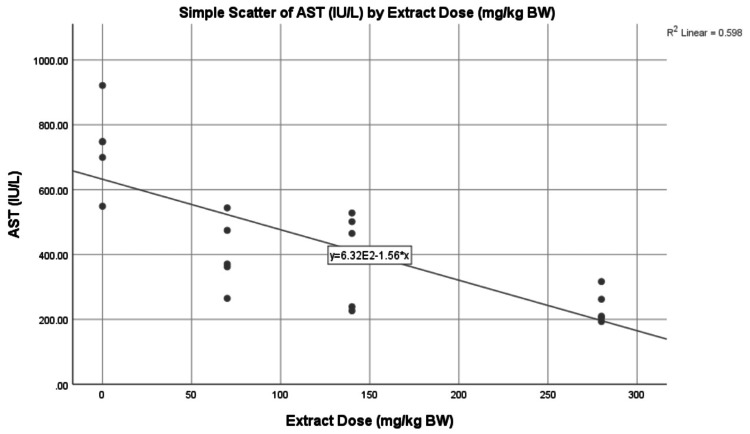
The correlation between extract doses and aspartate aminotransferase (AST) levels Scatterplot showing the negative correlation (p = 0.000; r = -0.774) between the dose of dried red jujube fruit extract and the AST levels.

The lowest average AST level was found in G1 with 174.78 ± 12.59 IU/L. The highest average AST level was found in G2 with 733.10 ± 133.16 IU/L. The AST levels in the treatment group formed a linear line between G1 and G2, sequentially from high to low, with an average value of G3 of 403.28 ± 108.11 IU/L, G4 of 392 ± 147.11 IU/L, and G5 of 237.30 ± 51.57 IU/L (Figure 3). The average AST level in G1 increased by 319.44% or +558.32 IU/L in G2, 130.74% or +228.50 IU/L in G3, 124.28% or +217.22 IU/L in G4, and 35.77% or +62.52 IU/L in G5. The average AST level in G2 decreased by 44.99% or -329.82 IU/L in G3, 46.53% or -341.10 IU/L in G4, and 67.63% or -495.80 IU/L in G5. The average AST level in G3 decreased by 2.80% or -11.28 IU/L in G4 and 41.16% or -165.98 IU/L in G5. The average AST level in G4 decreased by 39.46% or -154.70 IU/L in G5 (Table 4). There was a negative correlation between all extract doses and the AST levels, forming a linear line from top to bottom, as seen in Figure 4. A negative correlation means the higher the dried red jujube fruit extract doses, the lower the AST levels.

**Table 4:** Value difference and percentage of aspartate aminotransferase levels G1: negative control group; G2: positive control group; G3: treatment group 1; G4: treatment group 2; G5: treatment group 3; SD: standard deviation; IU/L: international unit/liter; +/↑: increase; -/↓: decrease.

Group	Value difference (IU/L)	Percentage
G1 → G2	+558.32	↑ 319.44%
G1 → G3	+228.50	↑ 130.74%
G1 → G4	+217.22	↑ 124.28%
G1 → G5	+62.52	↑ 35.77%
G2 → G3	-329.82	↓ 44.99%
G2 → G4	-341.10	↓ 46.53%
G2 → G5	-495.80	↓ 67.63%
G3 → G4	-11.28	↓ 2.80%
G3 → G5	-165.98	↓ 41.16%
G4 → G5	-154.70	↓ 39.46%

## Discussion

Extraction is a technique that separates bioactive compounds from raw materials in a sample [9]. The qualitative phytochemical screening revealed that dried red jujube fruit extract contained bioactive substances such as alkaloids, flavonoids, phenolics, saponins, tannins, and triterpenoids (Table 1). The extraction results of the maceration method are influenced by several factors, including the duration of maceration, the maceration temperature, the type and concentration of the dissolution, and the volume ratio between the sample and the dissolution [10].

The quantitative phytochemical analysis showed 6.52 mg GAE/g of total phenolic content and 1.92 mg RE/g of total flavonoid content contained in the dried red jujube fruit extract (Table 2). The TPC and TFC levels of dried red jujube fruit extract in this research were decreased compared to the fresh one in previous research, where the TPC was 23.96 mg GAE/g and the TFC was 7.07 mg RE/g [6]. These results revealed that the drying, packaging, and product storage processes contribute to the changes in the bioactive compounds of the jujube fruit extract.

The results of the quantitative phytochemical analysis in this research are in line with previous research, which showed that the drying process of jujube fruit causes a significant decrease in bioactive compound levels and antioxidant activities [11]. The reduction in TFC levels of jujube fruit extract is significantly affected by the duration and temperature of the drying process. The higher the temperature and the longer the drying process time, the lower the TFC levels of the jujube fruit extract [12]. The drying methods also affect the TPC and TFC levels, which are significantly decreased in the oven and sun drying methods [6]. The product storage factors, such as temperature and duration, also have a significant role in the reduction of bioactive compound levels in jujube fruit extract. TPC and TFC levels decreased faster at higher product storage temperatures and longer duration of product storage [13,14].

Acute hepatotoxicity in this research was conducted in vivo using a single dose of acetaminophen at 3 g/kg BW, and the aminotransferase levels were used as the indicator. The results shown in Figure 1, 3, revealed that a single dose of acetaminophen at 3 g/kg BW was a toxic dose that induced hepatotoxicity in Wistar rats. The increase of ALT and AST average levels from G1 to G2 occurred because a toxic dose of acetaminophen consumption caused an increase in N-acetyl-p-benzoquinone imine (NAPQI). The increase in the amount and speed of formation of NAPQI caused glutathione (GSH) to decrease drastically. GSH is an endogenous antioxidant in the human body, which could minimize hepatotoxicity by conjugating with NAPQI in sufficient amounts. GSH depletion caused NAPQI to prefer conjugating with hepatocytes, which could cause hepatocyte damage and extensive hepatocyte injury due to reactive oxygen species (ROS) production. Hepatocyte injury caused the release of ALT and AST, causing their levels to increase in the blood serum [15-18].

The doses of dried red jujube fruit extract used in the study were 70 mg/kg BW (G3), 140 mg/kg BW (G4), and 280 mg/kg BW (G5). The results in Figures 1, 3 showed that all doses of the dried red jujube fruit extract had a hepatoprotective effect on acetaminophen-induced acute hepatotoxicity. The decrease in the average ALT and AST levels from G2 to G3, G4, and G5 occurred because the dried red jujube fruit extract contains phenolics and flavonoids as exogenous antioxidants. Antioxidants minimize the risk of hepatocyte injury through a direct scavenging mechanism by directly donating electrons to free radicals. Flavonoids also inhibit the work of CYP450, resulting in a decrease in NAPQI production. Phenolics and flavonoids also increase endogenous antioxidants in the body [7,19,20].

The decrease in ALT and AST levels, along with increasing doses of dried red jujube fruit extract (Figures 2, 4), occurred because the higher the phenolic and flavonoid content, the higher the antioxidant activity. Antioxidants are essential in breaking down free radical chains. This theory is also in line with previous research, which proved that the hepatoprotective effect of fresh jujube fruit extract increases with increasing administration doses [7,21].

The ability of an extract to cause a specific effect in the smallest dose is referred to as extract potency. The average aminotransferase levels from G2 to G3, G4, and G5 experienced a significant decrease, where G3 was the treatment group with the lowest extract dose (Figures 1, 3). These results revealed that the potent dose of dried red jujube fruit extract to reduce ALT and AST levels are 70 mg/kg BW.

Efficacy is the ability or effectiveness of an extract to achieve a specific treatment target. The average aminotransferase levels from G2 to G3, G4, and G5 experienced a significant decrease, with the decrease in aminotransferase levels from group G2 to G5 having the highest percentage (Tables 3, 4). These results revealed that the 280 mg/kg BW dose has the highest efficacy in reducing the ALT and AST levels in acetaminophen-induced acute hepatotoxicity.

This research has provided evidence for the hepatoprotective effect of dried red jujube fruit extract. However, the mechanism still requires to be researched further through several other supporting specific parameters. Further studies and analyses will be needed to fully prove the protection mechanism of dried red jujube fruit extract on the liver against acetaminophen-induced acute hepatotoxicity, such as the histopathological examination of the liver, and the measurement of GSH levels, the glutathione peroxidase (GPx), malondialdehyde (MDA), superoxide dismutase (SOD), or catalase (CAT) activities.

## Conclusions

The dried red jujube fruit extract has a hepatoprotective effect on aminotransferase levels against acetaminophen-induced acute hepatotoxicity. There was a negative correlation between dried red jujube fruit extract at 70 mg/kg BW, 140 mg/kg BW, and 280 mg/kg BW with the aminotransferase levels.
